# Adherence to topical anti-glaucoma medications in Sidama regional state, Southern Ethiopia

**DOI:** 10.1371/journal.pone.0284200

**Published:** 2023-04-26

**Authors:** Mekete Wodajnew Melaku, Betelhem Temesgen Yibekal, Ketemaw Zewdu Demilew

**Affiliations:** 1 Department of Ophthalmology and Optometry, College of Medicine and Health Sciences, Hawassa University, Hawassa, Ethiopia; 2 Department of Optometry, College of Medicine and Health Sciences, University of Gondar, Gondar, Ethiopia; The University of Iowa, UNITED STATES

## Abstract

**Purpose:**

To assess the proportion of adherence to topical anti-glaucoma medications and associated factors among glaucoma patients in Sidama regional state, Ethiopia.

**Methods:**

An institution based cross-sectional study was conducted from May 30 to July 15, 2022 at Hawassa University comprehensive specialized and Yirgalem General Hospitals in Sidama regional state, Ethiopia. A systematic random sampling method was used to select 410 study participants. An adapted eight-item self-reported questionnaire was used to assess adherence. Binary logistic regression was used to identify factors associated with adherence to topical anti-glaucoma medications. Variables with a p-value of <0.05 under multivariable analysis were taken as statistically significant factors for adherence. The strength of the association was measured by using an adjusted odds ratio with 95% confidence interval.

**Results:**

A total of 410 participants were included giving a response rate of 98.3%. Those who were adherent to their medications were 221(53.9%) (95% CI: 48.8–58.5). Urban residence (AOR = 2.81: 95% CI = 1.34–5.87), higher educational level (AOR = 3.17: 95% CI = 1.24–8.09), monthly follow-up frequency (AOR = 3.30: 95% CI = 1.79–6.11) and normal vision (AOR = 6.58: 95% CI = 3.03–10.84) were significantly associated with adherence.

**Conclusion:**

More than half of glaucoma patients attending at Hawassa University comprehensive specialized and Yirgalem general hospitals were adherent to their topical anti-glaucoma medications. Urban residence, educational level, follow-up frequency, and normal vision were associated with adherence.

## Introduction

Glaucoma refers to a group of diseases in which optic nerve damage is the most common pathology that results in visual field defects, followed by vision loss. It is often asymptomatic in early stages for which high intraocular pressure (IOP) is a major and the only modifiable risk factor [1,2]. Medication adherence is the extent to which patients take medications as prescribed by their health care providers [3].

Glaucoma is the world’s second most prevalent blinding condition, and the visual loss in glaucoma is irreversible [4]. Lowering intraocular pressure (IOP) is the only proven strategy that minimizes the risk of glaucoma progression. Medical therapy, laser, and surgical treatment can be used to lower IOP, of which topical ocular hypotensive drops are the first line therapy for the majority of cases [5].

Topical anti-glaucoma medications are used for a long period of time and require long-term adherence to achieve a maximum IOP lowering effect [6,7]. Adherence to topical anti-glaucoma medications can considerably slow disease progression and visual disability at a lower cost [8,9]. Due to an increasing frequency of bad outcomes and greater costs of eye care services, non-adherence to glaucoma treatment is a challenge and a growing issue for health-care systems, clinicians, and other stakeholders to effectively treat glaucoma [2,8].

A systematic review conducted in Latin America revealed that adherence to topical anti-glaucoma medications varies from 4.6% to 59% [10], and in the United States of America, it was 51% [11]. The proportion of adherence was 72.6% in Saudi glaucoma patients [12]. In Ethiopia, adherence to topical anti-glaucoma medications ranges from 32.5% to 61.4% [13–16].

However, evidence-based information was lacking regarding the proportion of adherence to topical anti-glaucoma medications and associated factors in Southern Ethiopia, particularly in the study area. Therefore, this study will fill this gap by assessing adherence to topical anti-glaucoma medications and associated factors among adult glaucoma patients attending at two eye care centers (Hawassa University comprehensive specialized and Yirgalem general hospitals) in Southern Ethiopia. The result will be important for eye care service providers and policy makers in order to develop appropriate strategies to improve patients’ adherence to topical anti-glaucoma medications.

## Methods and materials

### Study design and period

A hospital based cross-sectional study was conducted from May 30 to July 15, 2022.

### Study area

The study was conducted at Hawassa University comprehensive specialized hospital (HUCSH) and Yirgalem general hospital (YGH). HUCSH is located in Hawassa city, which is the capital city of Sidama regional state. It is 273 km south of the capital of Ethiopia, Addis Ababa. Yirgalem general hospital (YGH) is a teaching hospital in Yirgalem city, which is 45 km south of Hawassa city.

### Source population

All glaucoma patients who attended at HUCSH and YGH were the source population.

### Study population

Glaucoma patients who attended the hospitals during the study period were the study population.

### Inclusion and exclusion criteria

#### Inclusion criteria

All patients aged ≥18 years who were diagnosed to have glaucoma or ocular hypertension and who were put on one or more topical anti-glaucoma medications in one or both eyes for at least 6 months before commencement of the study were included.

#### Exclusion criteria

Glaucoma patients treated with laser or surgical therapy and on post-operative medications, stable post laser or surgical therapies who were not on topical anti-glaucoma medications, and use of any non-glaucoma drops during the data collection period were excluded from the study.

### Sample size determination

The required sample size was determined using a single population proportion formula. By taking the proportion of adherence (56.2%) from a study done at Felege-Hiwot referral hospital in Ethiopia [14], margin of error 5%, z statistic at 95% confidence interval 1.96 and 10% non-response rate, the final sample was determined to be 417.

### Sampling procedures

The calculated sample size was proportionally allocated for the two hospitals based on the number of patients in each hospital which was 243 patients from HUCSH and 174 patients from YGH. Then a systematic random sampling method was used to select the study subjects from each hospital with a sampling fraction of two. During each data collection day, lottery method was used to determine whether to start with the first or second patient then every other patient is included in the study.

### Study variables

#### Dependent variable

Adherence to topical anti-glaucoma medications (Yes or No).

#### Independent variables

**Socio-demographic**: Age, sex, marital status, residence, educational level, occupation, knowledge about glaucoma, monthly income.

**Medication related:** Health insurance, family support, number of topical drops, type of topical drops, drug side effects, frequency of daily dose, medication availability and medication affordability.

**Glaucoma related:** Type of Glaucoma, stage of glaucoma, duration of glaucoma, visual acuity, follow-up frequency, intra-ocular pressure.

**Health care system related:** Information about medication, home to hospital distance.

### Operational definition

Adherence was assessed by using self-reported 8-item adherence scale questions; each correct response had a value of 1 and the incorrect response had 0. The overall proportion of adherence was categorized as adherent or non-adherent using median score value (5) as a cutoff point. Thus, participants who scored greater than or equal to 5 (the median) were categorized as adherent, whereas participants who scored less than the median value were categorized as non-adherent [14].

Knowledge: was assessed using 8 questions; each correct response had a value of 1, and the incorrect response had 0. The overall knowledge of the participants regarding glaucoma and its medication was categorized as ‘good’ or ‘poor’ using mean score value (4.1) as a cutoff point. Thus, participants who score greater than or equal to the mean value (4.1) were considered as having good knowledge, whereas participants who scored less than the mean value were considered as having poor knowledge [17].

Stages of glaucoma in the worse eye [1].

Early glaucoma: vertical cup to disc ratio (CDR): ≥0.5 to 0.7

Moderate glaucoma: vertical CDR: ≥0.7–0.85

Advanced glaucoma: vertical CDR ≥0.85–0.95

Absolute glaucoma: vertical CDR ≥0.95 with vision of no light perception (NLP)

Visual impairment: was defined as a presenting vision acuity of <6/12 in the better eye based on 11^th^ international classification of diseases (ICD 11^th^) definition of visual impairment. Visual impairment was also categorized as follows.

Normal: from 6/6 to 6/12

Mild VI: <6/12 to 6/18

Moderate VI: from 6/24 to 6/60

Sever VI: <6/60 to 3/60

Blind: <3/60

### Data collection tool and procedures

A structured interviewer-administered questionnaire and a data extraction format were used to collect data. A face-to-face interview was conducted to collect sociodemographic, medication related, and health care system-related data. The data collection tool was developed from different articles published on adherence to topical anti-glaucoma medications. We reviewed the tools in the papers and adapted our own tool. Then our data collection tool was consulted with experts on glaucoma management and researchers for the necessary modifications. Finally an adapted eight-item self-reported questionnaire was used to assess adherence [13,14]. Answers "no" were coded as 1, while "yes" as 0 except for question number 1, in which "yes" was coded as 1 and "no" was 0. The items were then summed to give a range of scores from 1 to a maximum score of seven. Participants who scored ≥5 on self-reported questions were categorized as adherent to their glaucoma medication. The data extraction format was used to collect glaucoma related data including types of glaucoma, stage of glaucoma, IOP, and visual acuity from medical cards. Two Optometrists from each hospital collected the data.

### Data quality control

The questionnaire was pretested on 5% of the sample size at Wachamo University Nigist Eleni Mohammed Comprehensive Specialized Hospital, Hosaena City, Southern Ethiopia, and the reliability of the tool was checked (Cronbach’s Alpha = 0.803). Before data collection, training was given to the data collectors. During data collection, there was daily supervision and discussions, and the collected data was evaluated for its completeness and consistency by the principal investigator and a supervisor. The English version of the questionnaire was translated into Amharic and then back-translated to English by language experts for consistency. Data was collected using the Amharic version of the questionnaire through face-to-face interviews.

### Data processing and analysis

After checking completeness, the data was entered and cleaned using epi-data version 3.1 and then exported to SPSS version 25 for analysis. Analysis was done by the investigator. Proportions, frequency, and summary statistics (median and interquartile range) were used to summarize the study variables. A binary logistic regression model was fitted to identify the possible predictors associated with the outcome variable. Variables with a p-value of less than 0.2 in the bivariable analysis were entered into a multivariable binary logistic regression. The enter method was used to select a significant variable and the fitness of the model was checked using Hosmer and Lemeshow goodness of fit (0.848). The strength of association between dependent and independent variables was expressed by using an adjusted odds ratio with a 95% confidence interval. A variable with a P-value of less than 0.05 was considered statistically significant.

### Ethical considerations

The study was conducted in accordance with the Declaration and tenets of Helsinki. Ethical clearance was obtained from the University of Gondar, College of Medicine and Health Sciences, School of Medicine ethical review committee. Permission was obtained from the two hospitals. Written informed consent was obtained from each participant after explaining the purpose of the study.

## Results

### Socio-demographic characteristics of the study participants

A total of 410 patients were included in this study and the response rate was 98.3%. The median age was 56 years, with an Interquartile Range (IQR) of 47–67.25 years. Out of the total study participants, 257 (63.2%) were males. The majority of study participants (329(80.2%) were married “Table 1”.

**Table 1:** Socio-demographic characteristics of the study participants (n = 410).

Variables	Frequency	Percent (%)
Age		
25–47	106	25.9
48–56	108	26.3
57–67	94	22.9
68–85	102	24.9
Sex		
Male	259	63.2
Female	151	36.8
Marital status		
Married	329	80.2
Single	81	19.8
Residence		
Urban	232	56.6
Rural	178	43.4
Educational level		
Can’t read and write	145	35.3
Can read and write	43	10.5
Elementary	66	16.1
High school	38	9.3
Diploma and above	118	28.8
Occupation		
Farmer	166	40.5
Employee	106	25.9
Merchant	66	16.1
Retired	41	10.0
Housewife	14	3.4
Others*	17	4.1
Knowledge		
Good	166	40.5
Poor	244	59.5
Monthly income(ETB)		
<846	103	25.1
846–1561	104	25.4
1562–4631	102	24.9
>4631	101	24.6

*priests, laborer, ETB-Ethiopian birr.

### Medication related factors

About three quarters 312 (76.1%) of the study participants were using a single medication with a twice-daily dose. Timolol eye drop was used by about two thirds 281(68.5%) of the study participants and 331(80.7%) study participants had no health insurance “Table 2”.

**Table 2:** Medication related factors of study participants (n = 410).

Variables	Frequency	Percent (%)
Number of medications		
One	312	76.1
Two	84	20.5
Three	14	3.4
Frequency of daily dose		
Twice	312	76.1
Three times and more	98	23.9
Name of medications		
Timolol	281	68.5
Other* with/without timolol	129	31.5
Side effect		
Yes	76	18.5
No	334	81.5
Availability		
Yes	120	29.3
No	290	70.7
Payment type		
Health insurance	79	19.3
Self-buy	331	80.7
Affordability		
Affordable	133	32.4
Unaffordable	277	67.6
Family support		
Yes	207	50.5
No	203	49.5

*dorzamol, pilocarpine, latanoprost, dorzolamide.

### Glaucoma related factors

Two thirds 280 (68.3%) of cases were primary open angle glaucoma (POAG) and nearly half of the total glaucoma cases 185 (45.1%) were advanced stage “Table 3”.

**Table 3:** Glaucoma related factors of study participants (n = 410).

Variables	Frequency	Percent (%)
Types of glaucoma		
POAG	280	68.3
PXG	111	27.1
Others*	19	4.6
Stage of glaucoma		
Early	77	18.8
Moderate	107	26.1
Advanced	185	45.1
Absolute	41	10.0
Duration of treatment(months)		
6–12	144	35.1
13–24	80	19.5
25–48	96	23.4
49–240	90	22.0
Intraocular pressure(mmHg)		
14–20	105	25.6
21–26	112	27.3
27–34	96	23.4
35–60	97	23.7
Follow-up frequency		
Monthly	186	45.4
Every two months	102	24.9
Every three months and above	122	29.7
Visual acuity		
Normal	156	38
Mild to Moderate VI	172	42
Sever VI and Blindness	82	20

*normal tension glaucoma, traumatic glaucoma, uveitic glaucoma, primary closed angle glaucoma, PXG = pseudoexfoliative glaucoma, VI = visual impairment, POAG = primary open angle glaucoma.

### Health care system related factors

More than two thirds 279 (68%) of the study participants obtained information about medication from only physician. The median distance of the hospitals from study participants’ homes was 50 kilometers, with an Interquartile Range (IQR = 23–150) “Table 4”.

**Table 4:** Health care related factors of study participants (n = 410).

Variables	Frequency	Percent (%)
Source of information about medication		
Only from physician	279	68.0
From both physician and pharmacist	131	32.0
Home to hospital distance (km)		
<24	103	25.1
24–50	116	28.3
51–150	97	23.7
>150	94	22.9

km = Kilometers.

### Proportion of adherence to topical anti-glaucoma medications

Assessment of participants’ responses to self-reported questions revealed that, 221 (53.9%) (95% CI: 48.8–58.5) were adherent to their topical anti-glaucoma medications.

### Factors affecting adherence to topical anti-glaucoma medications

All independent variables were tested for crude association with adherence to topical anti-glaucoma medications using bivariable logistic regression analysis and variables with a p-value less than 0.2 were entered into multivariable regression. Multivariable logistic regression analysis was used to fit age, gender, residence, educational status, duration of treatment, follow-up frequency, visual acuity, medication affordability, intraocular pressure and stage of glaucoma. Residence, educational status, follow-up frequency, and visual acuity were significantly associated with adherence in multivariable regression.

Urban residents were 2.81 (AOR = 2.81; 95% CI = 1.34–5.87) times more likely to be adherent than rural residents. Participants with higher educational level (diploma and above) were 3.17 (AOR = 3.17; 95% CI = 1.24–8.09) times more likely to be adherent than those who couldn’t read and write. The odds of being adherent to topical anti glaucoma medications among participants who had monthly follow-up frequency were 2.88 (AOR = 3.30; 95% CI = 1.79–6.11) times higher than those appointed every three months and above. Participants who had normal vision were 4.87(AOR = 6.58; 95% CI = 3.03–10.84) times more likely to be adherent to their topical anti-glaucoma medications than participants who were with severe visual impairment and blindness “Table 5”.

**Table 5:** Factors associated with adherence to topical anti-glaucoma medications (n = 410).

Variables	Adherent	Non-adherent	COR 95% CI	AOR 95% CI	P-value
**Age**					
25–47	79	27	3.42(1.90–6.15)	0.85(0.37–1.94)	
48–56	54	54	1.17(0.68–2.01)	0.57(0.27–1.19)	
57–67	41	53	0.91(0.52–1.59)	0.61(0.31–1.23)	
68–85	47	55	1.00	1.00	
**Sex**					
Male	148	111	1.43(0.95–2.13)	1.30(0.75–2.25)	
Female	73	78	1.00	1.00	
**Residence**					0.006
Urban	165	67	5.37(3.50–8.21)	2.81(1.34–5.87)	
Rural	56	122	1.00	1.00	
**Educational status**					0.007
Can’t read and write	47	98	1.00	1.00	
Can read and write	14	29	1.01(0.49–2.08)	0.56(0.23–1.38)	
Elementary	36	30	2.50(1.38–4.54)	1.19(0.51–2.77)	
High school	29	9	6.72(2.95–15.33)	2.90(0.92–9.11)	
Diploma and above	95	23	8.61(4.86–15.28)	3.17(1.24–8.09)	
**Medication affordability**					
Affordable	82	51	1.60(1.05–2.43)	1.72(.98–2.97)	
Unaffordable	139	138	1.00	1.00	
**Duration of treatment (months)**					0.095
6–12	87	57	2.77(1.60–4.78)	2.39(1.16–4.93)	
13–24	49	31	2.87(1.53–5.34)	1.93(0.86–4.31)	
25–48	53	43	2.23(1.24–4.03)	2.17(1.04–4.56)	
49–240	32	58	1.00	1.00	
**Follow up frequency**					<0.001
Monthly	125	61	3.63(2.25–5.87)	3.30(1.79–6.11)	
Every two months	52	50	1.84(1.08–3.15)	1.64(0.83–3.26)	
Every three months and above	44	78	1.00	1.00	
**Glaucoma severity**					
Early	55	22	4.33(1.94–9.69)	1.03 (0.36–2.91)	
Moderate	66	41	2.79(1.32–5.88)	0.99 (0.37–2.66)	
Advanced	85	100	1.47(0.73–2.96)	1.11 (0.46–2.64)	
Absolute	15	26	1.00	1.00	
**Visual acuity**					<0.001
Normal	122	34	8.67(4.72–15.94)	6.58(3.03–10.84)	
Mild and moderate VI	75	97	1.87 (1.06–3.28)	2.37 (1.20–4.68)	
Sever VI and blind	24	58	1.00	1.00	

## Discussion

The proportion of PXG (27.1%) in this study was high which is comparable to study done in Finland (26%). The reason for this increased proportion of PXG may be the older ages of our study participants. Around 75% of the study participants are older than 48years old. Old age is one of the risk factors of PXG [18].

An increase in adherence to topical anti-glaucoma medications is considered as an important factor for the better management of glaucoma. In this study, the proportion of adherence to topical anti-glaucoma medications was 53.9% (95% CI: 48.8–58.5). This result was consistent with a study conducted at Felege Hiwot hospital in Ethiopia (56.2%) [14].

The proportion of adherence in this study was higher than studies done at Jimma Hospital in Ethiopia, 32.5% [16], Menelik Hospital in Ethiopia 42.6% [15], Egypt, 46.4% [19] and Iran, 34.6% [20]. The reason for this difference might be due to differences in data collection tools, definition of adherence and sampling technique. This study used adapted 8-item self-report questions. However, studies done at Menelik hospital and in Iran used an Eight-item Morisky Medication Adherence Scale (MMAS-8) and 4-item Morisky Medication Adherence Scale (MMAS-4) data collection tools respectively. In this study patients were labeled adherent when their score was greater than or equal to the median (62.5%) score while the study done in Egypt defined non-adherent patient as one who omits over 10% of the weekly doses.

On the other hand, the proportion of adherence to topical anti-glaucoma medications in this study was lower than the studies conducted at University of Gondar Hospital Ethiopia, 61.4% [13], Saudi, 72.6% [12] and German, 66.5% [21]. The possible justification might be the differences in the study setting, socioeconomic and educational status of the study populations. The study conducted at University of Gondar hospital was single-centered, whereas the present study was conducted at two ophthalmic centers. All the study participants in German were insured by the public health insurance agency [21] unlike study participants in this study 79 (19.3%) which might be the reason for the difference. Being insured was reported to be a significant factor for good adherence [19]. All study participants of the study conducted in Saudi had educational background of higher than high school graduates [12]. Only 156(38%) of study participants had educational background of high school and above. The variations in educational background of the study subjects of the two studies might be the reason for the differences in proportion of adherence. Higher education is a known factor for good adherence [15].

According to this study, urban residents were found to be more adherent than rural residents. This was consistent with studies done in Ethiopia [13,14]. This might be because urban residents had good knowledge about glaucoma and the importance of adherence to topical anti-glaucoma medications [22]. Urban residents also have better access to eye care centers and pharmacies for replenishment when they run out of medications before their next visit.

In addition, patients with higher educational level (diploma and above) were more likely to be adherent compared to patients who can’t read and write. This result is supported by the study done in Ethiopia [15]. The reason for this may be because patients with higher educational level might easily understand the prescribed regimen [23] might have good knowledge about the importance of medication adherence [17,22,24] and may take reminder notes.

The present study also revealed that glaucoma patients with monthly follow-up visits were more likely to be adherent than those who came to the hospitals less frequently (every three months and more). This was supported by similar studies done in Iran and Ethiopia [16,20]. This might be explained by the fact that patients with frequent follow-ups might get frequent reinforcements from their physicians and frequent medication refills, which might keep them adherent to their medications. Additionally, patients with frequent follow-ups might learn more about the importance of adherence to glaucoma medications [20] from their physicians or from other patients.

Finally, this study also confirmed that glaucoma patients with normal vision were more adherent to their topical anti-glaucoma medications than those with severe visual impairment and blindness. This was supported by studies done in Ethiopia [13,14] and Brazil [25]. The reason might be due to the fact that patients with normal vision might have less difficulty identifying and applying their drops than those with severe visual impairment and blindness. In other words, the reason for poor vision might be poor adherence to topical anti-glaucoma medications. Another possible reason could be that patients with good vision might be economically better, which might be helpful to get a continuous supply of topical anti-glaucoma medications [16].

In this study around 45% of participants were non adherent to their topical anti-glaucoma medications. Therefore, appropriate and frequent patient education on the lifelong nature of the disease and the importance of keeping with medication plans may improve patients’ adherence to their medication.

## Conclusion

More than half of glaucoma patients attending at Hawassa University comprehensive specialized and Yirgalem general hospitals were adherent to their topical anti-glaucoma medications. Urban residency, higher educational level, monthly follow-up frequency and normal visual acuity were significantly associated with adherence.

## Supporting information

S1 Data (SAV)
